# A Qualitative Study of Quality of Life Concerns following a Melanoma Diagnosis

**DOI:** 10.1155/2017/2041872

**Published:** 2017-05-28

**Authors:** Rachel I. Vogel, Lori G. Strayer, Rehana L. Ahmed, Anne Blaes, DeAnn Lazovich

**Affiliations:** ^1^Masonic Cancer Center, University of Minnesota, Minneapolis, MN, USA; ^2^Division of Gynecologic Oncology, University of Minnesota, Minneapolis, MN, USA; ^3^Department of Dermatology, University of Minnesota, Minneapolis, MN, USA; ^4^Department of Medicine, Division of Hematology and Oncology, University of Minnesota, Minneapolis, MN, USA; ^5^Division of Epidemiology and Community Health, University of Minnesota, Minneapolis, MN, USA

## Abstract

The goal of this study was to identify a relevant and inclusive list of quality of life issues among long-term survivors of melanoma. Individuals diagnosed with stage I-III cutaneous melanoma and had survived 1-5 years, ages 18-65 years at diagnosis, were recruited. Five focus groups were conducted with 33 participants in total. Discussions centered on participants' experiences at diagnosis, as well as ongoing physical, emotional, and social concerns, and behavioral changes since diagnosis. The majority of participants reported shock, fear, and feeling overwhelmed at the time of diagnosis. Some reported lingering physical concerns, including pain, numbness, and lymphedema, while a few reported no lasting issues. Emotional concerns were common, with most reporting anxiety. Several also noted feeling lonely and isolated. Social concerns included alteration of activities to avoid sun exposure, issues with family communication, and frustration with the lack of appreciation of the seriousness of melanoma by others. Finally, while many participants reported changes to their sun exposure and UV-protection behaviors, some reported little to no change. The shared experiences among participants in this study confirm the unique nature of melanoma and the need for interventions designed to improve the health and quality of life of melanoma survivors.

## 1. Background

Melanoma, one of the most serious types of skin cancer, is unlike most common cancer types in that the incidence has been increasing over the past 30 years [1–3]. With a 5-year survival rate of 91%, there are currently over one million melanoma survivors in the United States [4]. Melanoma can be aggressive and resistant to treatment and can have the ability to metastasize even at the earliest stages [5]. These factors, and the young age at diagnosis, lead to an estimated 15–20 years of potential life lost [6], ranking seventh in number of years lost among all cancers in the United States [7]. A better understanding of the long-term and late effects of a melanoma diagnosis is needed as clinicians and researchers develop appropriate follow-up care guidelines and create educational and other interventions aimed at improving the lifespan and quality of life (QOL) of melanoma survivors.

QOL is a multidimensional concept that incorporates physical, psychological, and social functioning; an individuals' overall life satisfaction, perceptions of their health status, and ability to take part in valued activities are important components. To date, a limited number of studies examining QOL issues have been carried out in long-term melanoma survivors. The majority of these studies relied on currently available generic and cancer-specific instruments to assess a narrow range of issues, primarily related to emotional distress, anxiety, depression and psychosocial adjustment, or overall QOL [8–12].

The objective of the research reported here was to conduct focus groups to identify a relevant and inclusive list of QOL issues among melanoma survivors. Instead of focusing solely on the period of time immediately surrounding melanoma diagnosis and treatment, as others have done, we were interested in studying the issues survivors face as they live beyond their treatment.

## 2. Methods

### 2.1. Study Participants and Recruitment

The study was approved by the University of Minnesota Institutional Review Board (1201M09423). A convenience sample of patients treated by dermatologists and oncologists at the University of Minnesota were identified for participation in this study. English speaking individuals diagnosed with cutaneous melanoma in Minnesota since 2008, ages of 18–65 years at diagnosis, were eligible for this study. The age limits were imposed to match the eligibility criteria of a previous study conducted by the Principal Investigator (PI, Lazovich) [13]. Eligible individuals were sent an invitation letter, cosigned by their dermatologist, oncologist, or surgeon, and the PI. Potential participants were called to see if they were willing to participate in a focus group. A reminder letter was sent including a consent form to review prior to arrival.

### 2.2. Focus Groups

Focus groups were conducted to identify survivorship issues faced by people diagnosed with stage I–III melanoma. Groups were conducted separately for those with early stage disease (I and II) and advanced stage disease (III). Due to previous reports of differences in psychological adjustment after a diagnosis of melanoma between genders [9], focus groups were also held separately for men and women when possible. Five groups were formed from 33 participants, consisting of two all-male early stage melanoma groups (*n* = 6 and 7), two all-female early stage melanoma groups (*n* = 8 and 7), and one mixed-gender advanced stage melanoma group (*n* = 5).

The 90-minute focus groups were held at the University of Minnesota, Minneapolis campus. Participants provided written informed consent immediately prior to the start of the discussion. Each session was digitally audio-recorded and participants received $75 for their participation. All groups had the same moderator and comoderator and followed established methods for conduct of focus groups [14].

The question guide for the focus groups was developed iteratively. First, an extensive review was conducted of the literature that included published reports of QOL in melanoma survivors. The data from these studies were used to generate a list of topics thought to be important among persons diagnosed with melanoma. These topics were reviewed by all investigators and then refined into broad questions to serve as the focus group moderator guide [15]. The final questions included are presented in Table 1 and related to experience at diagnosis, physical, emotional, and social concerns since diagnosis, and behavioral changes since diagnosis.

At the end of each session, the moderator summarized key points discussed during the focus group and requested feedback from the group regarding the accuracy of the summary. All recordings from the focus groups were transcribed.

### 2.3. Analysis

We used standard procedures of qualitative thematic text analysis to analyze the focus group transcripts [15]. Two researchers (DL and RIV) independently read the transcripts and agreed to broad themes from the focus group discussions. Each researcher then conducted an analysis using descriptive coding techniques [14]. Results were compared for consistency and thoroughness and overarching themes and subtopics were agreed upon. Notes taken during the focus group discussions were used to complement the conclusions. Exemplary quotes from participants are provided as appropriate and presented verbatim.

## 3. Results

**Figure 1:**
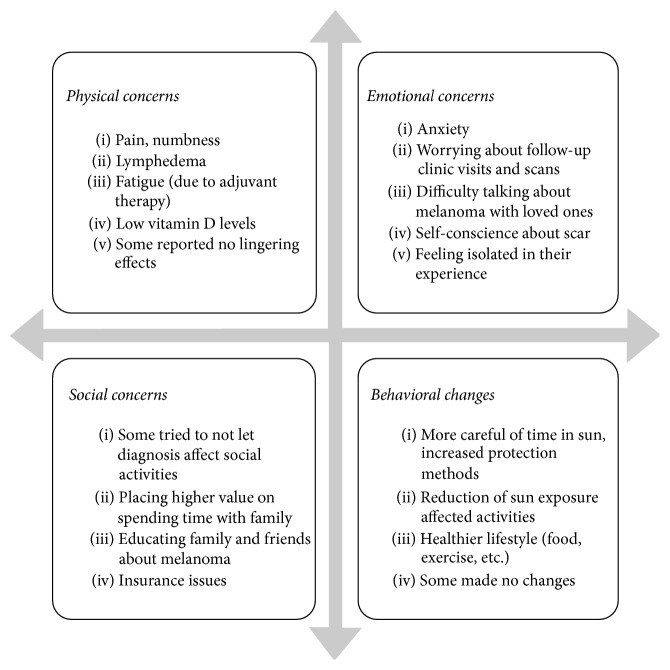


We identified 105 eligible melanoma patients, of whom 72 (68.6%) indicated they were willing to participate; 33 ultimately participated (31.4%). Among those who expressed initial interest but did not participate, the most common reason given was a scheduling conflict. Those who participated were similar to those who did not participate in gender and age. Participants were evenly distributed by gender (54.5% female), and most had a history of stage I disease (63.6%) with 18.2% each with a history of either stage II or stage III disease; the mean age was 49 ± 13 years. Approximately 25% had a family history of melanoma. A summary of the overarching themes is presented in Figure 1; details and quotes from the focus groups follow.

### 3.1. Experience at Diagnosis

Each focus group started with a discussion of what participants experienced at the time of the melanoma diagnosis. The large majority of participants reported feeling shock, fear, and disbelief of the severity of the diagnosis.

Fear, screaming. It's a word [cancer] you don't ever want to hear. That was my initial reaction.

A few said they had noticed changes in their skin but that their moles were assessed as normal by healthcare providers. They expressed anger when they finally received the diagnosis of melanoma.

I was angry […] I had been questioning this spot for a year and had been told it was nothing and not to worry about it. So learning that it was melanoma, I was angry.

Once diagnosed, most reported having surgery within a few days. Participants said the rapid pace of treatment was overwhelming as they tried to process the diagnosis, learn about melanoma, and undergo surgery. Many admitted they had little knowledge about melanoma before their diagnosis, which made the process more difficult. Those with early stage disease reported being thankful that their disease was caught early; however those with stage II and III disease described difficulty in making decisions regarding additional therapy.

Some described their melanoma diagnosis as a devastating and traumatic experience. In particular, those with younger children reported having concerns about their families and how family members would process the information.

I was sure that, at the time, this is it, I'm dying, this is it. I was absolutely petrified and absolutely scared to death.

In contrast, while not the majority, a few reported their diagnosis was not particularly concerning, neither at the time of diagnosis nor in survivorship.

I feel like I'm in the wrong group because mine was no big deal.

### 3.2. Physical Concerns

Next we asked about physical concerns, both at the time of diagnosis and in the survivorship period. Some participants reported lingering physical concerns, including pain, numbness, and lymphedema.

My melanoma was on the side of my face. So they went in to the neck resection and they went so deep that I was in a lot of pain … I still have neck pain on that side of my neck.

I wear compression garments on usually a daily basis to keep all that fluid from moving everywhere.

A few noted some restrictions and limited motion during recovery that resolved with time and/or physical therapy. The few participants who received adjuvant therapy reported additional side effects, with fatigue described as the most troublesome and pervasive.

A few with stage I disease and small tumors reported minor surgeries and no physical changes of note.

I didn't have any problem. I walked out of the doctor's office and went back to work. That's it.

Lastly, some reported requiring extra vitamin D supplementation and a few noted still struggling with low levels and the resulting fatigue that they attributed to sun avoidance.

### 3.3. Emotional Concerns

The majority of participants reported longer-term concerns that were emotional in nature, with most experiencing some form of anxiety. A few reported general anxiety, whereas others were worried about follow-up checks and tests and their general health and were fearful of other cancers and melanoma for themselves and their children. Some reported that their anxiety improved with time, while for others the anxiety remained years after their diagnosis.

You listen to every one of us, the anxiety is the one thing that really got us. It's the anxiety, and the anxiety continues.

… you get really uptight just before that scan. I don't even realize but my husband says, ‘you're so bad the week before you scan'.

A number of participants said they were surprised by the size of their scar and were self-conscious about it and the changes to their self-image.

It was just a teeny, tiny spot and they cut all the way down to the muscle and took all the tissue just for preventive.

When they ended up taking that piece out of my cheek … I look in the mirror and I don't see my normal face.

Many reported relying on their family for emotional support, whereas a few participants relied on faith and spirituality.

Some participants, however, reported difficulty discussing melanoma with their family.

I wish I could talk more about it because, I tell you, it's almost a taboo subject in my family. They just don't want to hear about it. It's fear or something.

Several participants also noted feeling lonely and isolated despite family support. Interestingly, this issue came up not during the discussion of emotional health but at the end of the focus groups when we asked subjects about their motivation for participating in our study. We consistently heard how appreciative they were to participate in the discussion group as an opportunity to meet with others diagnosed with melanoma.

… it was kind of nice to be able to leave here today and not feel alone. Because that was my biggest thing, I constantly felt alone. Like I was the only person who had it ….

### 3.4. Social Concerns

When asked about any social changes, participants noted a number of social concerns, including altering social activities to avoid sun exposure and wanting to educate others about melanoma. Most expressed a desire to not let their diagnosis affect their interactions; however some were careful to limit activities in the sun.

Some reported feeling the need to educate others about melanoma and prevention strategies. In particular, many reported being frustrated by the lack of appreciation by others of the seriousness of melanoma.

I was amazed by the number of people who hear melanoma, and they're like, oh that's skin cancer, not a big deal.

A few reported not having insurance or having a high deductible plan and therefore faced difficulties with financial uncertainty and receiving optimal care. Quite a few also stated trouble obtaining life insurance after the diagnosis.

The reason I never got the mole taken off [earlier] was because I didn't have insurance initially.

### 3.5. Behavioral Changes

When asked about any changes in health behaviors since diagnosis, many participants reported behavior changes. Some were limiting their time in the sun and increasing the use of protective clothing and sunscreen, particularly those with higher stage disease.

I don't see the sun.

I have a lot of those long-sleeve shirts and it will be 86 degrees outside and I'll put on this long-sleeve shirt and I'm just sweating. But I wear them.

Others, however, reported little to no change in their sun protection methods.

Probably not the right thing to do, but I very seldom use sunscreen.

In addition, some reported adopting a healthier lifestyle, including losing weight, exercising more, and reducing work load. A few others also reported placing higher value on family and that their family became closer.

While most followed their physician's recommendations regarding surveillance, a few reported ignoring or postponing check-ups, particularly after the first year or two.

### 3.6. Identity as Cancer Survivor

As our goal was not only to summarize the experience of those diagnosed with melanoma but also to understand how future researchers might approach them, we asked participants whether they considered themselves to be cancer survivors. The responses were approximately evenly split between yes and no, but the explanations were diverse. All with stage III disease self-identified as cancer survivors, both indicating the literal stance that melanoma is cancer and also noting that it is a very serious cancer.

I have a close friend that has breast cancer … she was doing regular chemo and so I know all of that is just as grueling. But I don't think people realize sometimes that the stuff we do is just as grueling or it's the same thing. Cancer is cancer.

Some with early stage disease, when asked about being a cancer survivor, said yes as well, though a few participants noted they felt there was a spectrum of cancer survivorship.

If you took a level of cancer survivor I would consider myself a one compared to a ten.

Participants who responded in the negative came from two different philosophies. Some did not consider melanoma serious enough or felt it was inappropriate because they did not have chemotherapy or radiation.

I don't want to downplay melanoma, but when you say cancer survivor … that's … I don't want to upset anybody, but I really don't consider melanoma like pancreatic cancer or breast cancer.

I feel like I didn't pay the full due with the chemo and the radiation and everything.

A few others, however, did not like the term because they felt they could develop another cancer.

I've had cancer twice. I've gone through it twice, I've survived it twice. I don't consider myself a cancer survivor. I won't consider myself a cancer survivor until somebody looks me in the face and says, you know what honey, you're never going to get it again.

## 4. Discussion

The objective of the study was to document the experiences of persons diagnosed with melanoma further from their diagnosis using focus groups. The experiences shared by participants in this study highlight a number of QOL issues facing melanoma survivors. QOL among melanoma survivors has been an active area of research over the past few years and our study compliments the results of others, confirming many of the same themes.

As previously reported, most participants reported being shocked and overwhelmed at the time of diagnosis and having significant information needs [16–18]. Our data also support those of others who found melanoma survivors may experience treatment-specific symptoms such as fatigue and nausea, associated with adjuvant therapy [19–21], and lingering altered sensation and pain at the surgery site [22]. In addition, lymphedema was reported by a number of participants and resulted in notable complications, as reported by others with melanoma [16]. As important, some participants reported no physical concerns.

A systematic review found that most issues facing melanoma survivors are psychological in nature [12]. As noted by others, anxiety was the main concern of participants in this study, though it manifested in numerous ways: general anxiety, fear of recurrence or of a new cancer, and fear of family members getting cancer [23]. For most, the anxiety was worst around time of diagnosis and decreased over time. Similar to other reports, participants also reported concerns about their surgical scars, including feelings of disfigurement and discomfort with comments about the appearance of their scar by others [16, 17, 24]. We heard these concerns most notably among those with head or neck tumors and those with scars that were harder to disguise with clothing. The experiences shared by these survivors suggest that healthcare providers should inquire about emotional health when conducting surveillance exams and provide approaches for coping with anxiety.

As previously reported, for some participants their melanoma diagnosis had a positive impact on their life because it strengthened family relationships [10], whereas others felt socially isolated when they felt they could not join others in outdoor activities involving sun exposure [25]. We were, however, surprised by the level of isolation and loneliness reported despite having family support. This high level of isolation was also recently reported by Oliveria et al. [25]. Upon probing, it appeared to be caused by the lack of public awareness about the seriousness of melanoma. Compared to breast cancer survivors, for example, who have many opportunities to network with and meet other survivors, a need exists to increase both awareness of melanoma and chances for melanoma survivors to interact and support each other. Healthcare providers and advocates might consider promoting the creation of support groups and online forums for melanoma survivors, in addition to further educating the public about the serious nature of melanoma.

Studies to date on sun exposure and protection behaviors among melanoma survivors have been mixed; some have reported most survivors being conscious of sun exposure and protection [25–30], whereas others report risky sun behaviors despite their diagnosis [11, 30, 31]. The participants in this study suggest that both are true; while many alter their behaviors to avoid sun exposure, some report not changing their behavior. These data suggest at least some melanoma survivors need additional education and support regarding appropriate sun exposure and protection habits to reduce their risk of future melanomas.

Finally, participants were divided on whether they considered themselves a “cancer survivor.” Similar responses have been reported in other cancers, including breast, prostate, colorectal, and hematologic cancers [32–37]. While most studies found the majority of survivors identify as being a cancer survivor, a significant proportion do not. Previous studies in other cancers have reported that those who do not endorse the term stated concerns about the cancer not being severe enough or the future being uncertain, particularly among those with a high risk of recurrence [33, 34, 38]. Due to the complexity of this issue, we encourage cautious use of the term “cancer survivor” when working directly with those who have been diagnosed with melanoma and instead recommend “person diagnosed with melanoma” until further research on the preferences of this population can be conducted.

Based on previous literature available at the time of the focus groups, we expected potential differences in experiences based on gender and wanted to ensure participant felt comfortable to share all concerns. In general, however, gender did not appear to drive differences in the experiences in our focus groups as much as disease stage and perceived seriousness of melanoma did. The numbers of groups were too limited to draw specific conclusions regarding gender differences.

The main strength of this study is the use of qualitative methods. Collecting data using focus groups allowed for a detailed and in-depth assessment of melanoma survivorship issues. These methods particularly permitted for capture of experiential personal information. We also focused on survivors of early stage disease, in contrast with most previous research. This study is not without limitations, however. The participants were one to four years from diagnosis and were from a single institution, an academic medical center. In addition few participants received adjuvant therapy. Therefore, the results here may not be generalizable to all melanoma survivors. Further, a portion of melanoma survivors had a family history of melanoma but this was not formally addressed during the focus groups and may have affected their concerns. In addition, qualitative research can be difficult to summarize in an objective way and despite efforts otherwise, our presence during data collection (i.e., conduct of focus groups) may have affected the subjects' responses.

## 5. Conclusions

With melanoma incidence on the rise and the unique nature of the disease in terms of both its potential early age of onset and severity, understanding the experiences of melanoma survivors is necessary from a public health perspective to promote optimal health and QOL in this growing population. Melanoma survivors participating in this study reported a diverse set of physical, emotional, and social concerns following diagnosis. The level of isolation and loneliness reported underscores the need to increase both awareness of melanoma and chances for melanoma survivors to interact and support each other. The shared experiences among participants in this study confirm the unique nature of melanoma and the need for interventions designed to improve the health and QOL of melanoma survivors.

## Figures and Tables

**Table 1:** Questions asked during focus groups.

(i) Think back to the time of your melanoma diagnosis.
(a) What ran through your mind?
(b) Was there anything particularly difficult or surprising about the surgery/treatment?
(ii) What changes have you noticed since diagnosis?
(a) Physical.
(b) Emotional.
(c) Social.
(iii) Have you changed any behaviors since your diagnosis?
(iv) Was there a silver lining to this experience for you?
(v) Do you consider yourself a cancer survivor?

## Abbreviations

QOL: Quality of life
PI: Principal investigator.

## Data Access

The data from the current study are available from the corresponding author on reasonable request.
